# Characteristics of metastasis and survival between male and female breast cancer with different molecular subtypes: A population‐based observational study

**DOI:** 10.1002/cam4.4469

**Published:** 2021-12-12

**Authors:** Wentong Fang, Yue Huang, Xu Han, Jinghui Peng, Mingjie Zheng

**Affiliations:** ^1^ Department of Pharmacy The First Affiliated Hospital Nanjing Medical University Nanjing China; ^2^ Department of Breast Surgery The First Affiliated Hospital Nanjing Medical University Nanjing China

**Keywords:** male breast cancer, metastatic patterns, molecular subtypes, prognostic outcomes

## Abstract

**Objective:**

Male breast cancer (BC) is a rare disease, having different clinicopathological features and survival outcomes from female patients. The aim of this research was to, combine with molecular subtypes, analyze the metastatic patterns, and prognosis between male and female patients, and to determine whether the gender was the independent prognostic factor for BC.

**Methods:**

Data used in this study were acquired from the SEER database from 2010 to 2016. The clinicopathology features and metastatic patterns were compared by the Chi‐square test and Fisher's exact test. Kaplan–Meier method was performed to compare overall survival (OS) and factors correlated with OS were determined by Cox regression models. Competing risk models were used to ascertain factors related to breast cancer‐specific death (BCSD).

**Results:**

Compared with female BC, the incidence of regional LN (HR 1.849, 95% CI 1.674–2.043, *p* < 0.001) and distant metastasis (HR 1.421, 95%CI: 1.157–1.744, *p* < 0.001) was higher in male BC. For regional LN metastasis, hormone receptor (HoR)−/HER2+ subtype occupied the majority in both male (55.56%) and female (36.86%) groups. For distant metastasis, HoR−/HER2− subtype (21.26%), and HoR−/HER2+ (7.67%) were in major in male and female group separately. Male patients shared similar combinations of metastases with female groups as for single‐site, bi‐site, and tri‐site metastasis. Gender was an independent prognostic factor for OS (*p* < 0.001) but not for BCSD(*p* = 0.620). In subgroup of patients with HoR+/HER2−(OS: *p *= 0.003; BCSD: *p* = 0.606), HoR+/HER2+(OS: *p *= 0.003; BCSD: *p* = 0.277), regional LN positive(OS: *p* = 0.005; BCSD: *p* = 0.379), or bone metastasis (OS: *p* = 0.030; BCSD: *p* = 0.862), the male cohort had poorer OS but similar BCSD with female cohort.

**Conclusions:**

Compared with female patients, male BC had different metastasis patterns and prognostic outcomes, and the affection of breast subtypes on metastasis and survivorship was also different. More attention needs to be paid for specific molecular subtype and more personalized therapeutic strategies should be customized while treating male patients.

## Graphical abstract

Male BC had different metastasis patterns and prognostic outcomes compared with female patients. The affection of breast molecular subtypes on metastasis and survivorship in male and female cohorts was different. More attention needs to be paid for specific molecular subtype and more personalized therapeutic strategies should be customized while treating male patients.

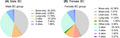

## 1 INTRODUCTION

Male breast cancer (BC) is a rare disease which is not fully studied.^1^, ^2^ The latest data from the American Cancer Society showed that male BC comprises about 0.94% of morbidity and 1.22% of mortality in all BC.^3^ According to previous studies on the basis of the Surveillance, Epidemiology, and End results (SEER) database, because of poor cognition of the disease and delayed diagnosis^1^, ^4^ ^,^ male patients are diagnosed with higher‐stage tumors and have a worse prognosis compared with female patients.^5^ The occurrence of lymph node and distant metastasis signifys poorer prognosis, as 90% of BC death is resulted from metastasis which is related to treatment failure.^6^ Thus, dig deep into metastatic patterns is beneficial for a better understanding of prognosis differences between male and female patients.

BC is defined into different molecular subtypes by biological markers.^7^ Different subtypes correlate with differing propensity to specific organs metastasis.^8^, ^9^, ^10^ It has been reported that, in female patients, human epidermal growth factor receptor 2 (HER2) positive subtype had a higher probability of liver metastasis, while lung metastasis was more commonly observed in the hormone receptor (HoR) negative/HER2 negative subtype.^8^, ^11^ As for male BC, the correlation between cancer subtypes and site‐specific metastasis patterns remains poorly understood. Besides, HER2 status^12^ and distant solid organ metastasis data^13^ were not specifically interpreted before 2010 in SEER database. Absent and incompatible data from retrospective registration studies limit definitive conclusions about HER2 status, solid organ metastasis patterns, and metastasis‐related survivorship of male BC.

Therefore, in our research, we excluded BC patients diagnosed before 2010 and incorporated BC data between 2010 and 2016 from the SEER database. Combined with BC molecular subtype, we horizontally and longitudinally studied metastatic BC patients to clarify different metastatic patterns and its effect on survivorship between male BC and female BC patients. Meanwhile, separately elaborating overall survival (OS) and breast cancer‐specific survival (BCSD) and determine whether gender was an independent prognostic factor for male BC.

## 2 METHODS

### 2.1 Study design

Data used in this study were obtained from the SEER program 1975 to 2016 Research Plus Additional Custom Treatment Data (www.seer.cancer.gov). HER2 status wasss not recorded before 2010, so we excluded BC patients diagnosed before 2010. In total, 252,473 BC patients were enrolled in the SEER database from 2010 to 2016.

The clinical inclusion criteria were as follows: (1) site recode was breast; (2) primary site was C50.0–50.9; (3) diagnostic confirmation was positive histological/clinical/visual/laboratorial/microscopic/radiographic diagnosis; (4) Type of follow‐up was active follow‐ up; (5) survival month was not less than 1 month; (5) BC was classified into four categories: HoR−/HER2−, HoR−/HER2+, HoR+/HER2−, or HoR+/HER2+.^12^ The process of patient selection is diagrammatized in Figure S1. Patients were divided into male BC and female BC group.

### 2.2 Variable classification

Patient characteristics included gender, age at diagnosis, race, and marital status. Tumor characteristics included laterality, grade, AJCC TNM stage (adjusted 8th edition),^14^ histology, regional lymph node (LN), bone metastasis, brain metastasis, liver metastasis, lung metastasis, distant LN metastasis, other distant metastasis, molecular subtype, surgery, chemotherapy, and radiation therapy. All metastasis variables referred to metastases which were identified at time of diagnosis. Because the SEER database was unable to distinguish between individuals that truly did not receive chemotherapy and those for whom this data were missing, we classified them into one category to differentiate them from patients who received the chemotherapy. OS and BCSD were used as outcome characteristic.

### 2.3 Statistical analysis

Variables in male BC and female BC group were conversed to categorical variable and compared by the Chi‐square test^15^ and Fisher's exact test.^16^ Multivariate analysis was performed by logistic regression. Kaplan–Meier survivor function^17^ was performed to compare OS and Cox regression models^18^ were used to determine factors associated with OS. Nelson–Aalen cumulative hazard function^19^ and competing risk models^20^ were used to determine factors associated with BCSD. Subgroup analyses were performed according molecular subtype and metastasis site. Propensity score matching (PSM) analysis was performed based on age, race, marital status, grade, laterality, AJCC stage, subtype, surgery, radiation, chemotherapy at a 1:1 ratio to adjust for the differences among the male BC and female BC groups.^21^ Stata 13.0 was performed for survival analysis (Stata Corp, College Station, TX, USA), and SPSS 23.0 was conducted for other analysis (SPSS Inc. Chicago, IL, USA).^22^, ^23^

## 3 RESULTS

### 3.1 Patient characteristics

From 2010 to 2016, 227,121 BC patients met the inclusion criteria and were enrolled in our research, including 1704 male BC and 22,5417 female BC patients (Table 1). Parameters including race, marital status, histology, grade, AJCC T classification, AJCC N classification, AJCC M classification, molecular subtype, chemotherapy, and radiation therapy showed significantly differences between the two cohorts. Compared with female BC group, male BC group tend to have older age, higher rate of black race, married status, ductal histology, HoR positive, poorer tumor differentiation, and later TNM stage (*p* < 0.05). In terms of therapies, fewer male BC patients received chemotherapy and radiation therapy than female BC patients (*p* < 0.05).

**TABLE 1:** Baseline clinical characteristics of male BC and female BC in the SEER database

Characteristics	Male BC (*n* = 1704)	Female BC (*n* = 2,25,417)	*p* values
Age			
<=60	463	104,019	*p *< 0.001
60–70	516	60,453	
>70	725	60,945	
Race			
White	1334	175,284	*p *< 0.001
Black	242	21,584	
Others	128	28,549	
Laterality			
Right	808	111,250	0.265
Left	892	113,737	
Other	4	430	
Marital status			
Married	1102	121,996	*p *< 0.001
Unmarried	514	91,911	
Unknown	88	11,510	
Histology			
Ductal	1522	176,067	*p *< 0.001
Lobular	20	23,210	
Other	162	26,140	
Grade			
Ⅰ	210	52,697	*p *< 0.001
Ⅱ	865	96,686	
Ⅲ/IV	558	67,442	
Unknown	71	8592	
AJCC stage			
Ⅰ	501	99,013	*p *< 0.001
Ⅱ	600	63,370	
Ⅲ	238	20,920	
IV	101	9245	
Other	264	32,869	
AJCC T classification			
T1	658	114,594	*p *< 0.001
T2	608	57,314	
T3	40	12,045	
T4	115	7467	
Other	283	33,997	
AJCC N classification			
N0	827	133,604	*p *< 0.001
N1	430	44,347	
N2	123	9706	
N3	76	6247	
Unknown	248	31,513	
AJCC M classification			
M0	1372	187,350	*p *< 0.001
M1	101	9245	
	231	28,822	
Molecular subtype			
HoR+/HER2‐	1461	167,383	*p *< 0.001
HoR+/HER2+	188	23,516	
HoR−/HER2+	18	10,096	
HoR−/HER2−	37	24,422	
Surgery			
Yes	1544	206,849	0.085
No/unknown	160	18,568	
Chemotherapy			
Yes	620	87,715	0.033
No/unknown	1084	137,702	
Radiation therapy			
Yes	484	111,337	*p *< 0.001
No/unknown	1220	114,080	

### 3.2 Regional LN metastasis

**FIGURE 1:**
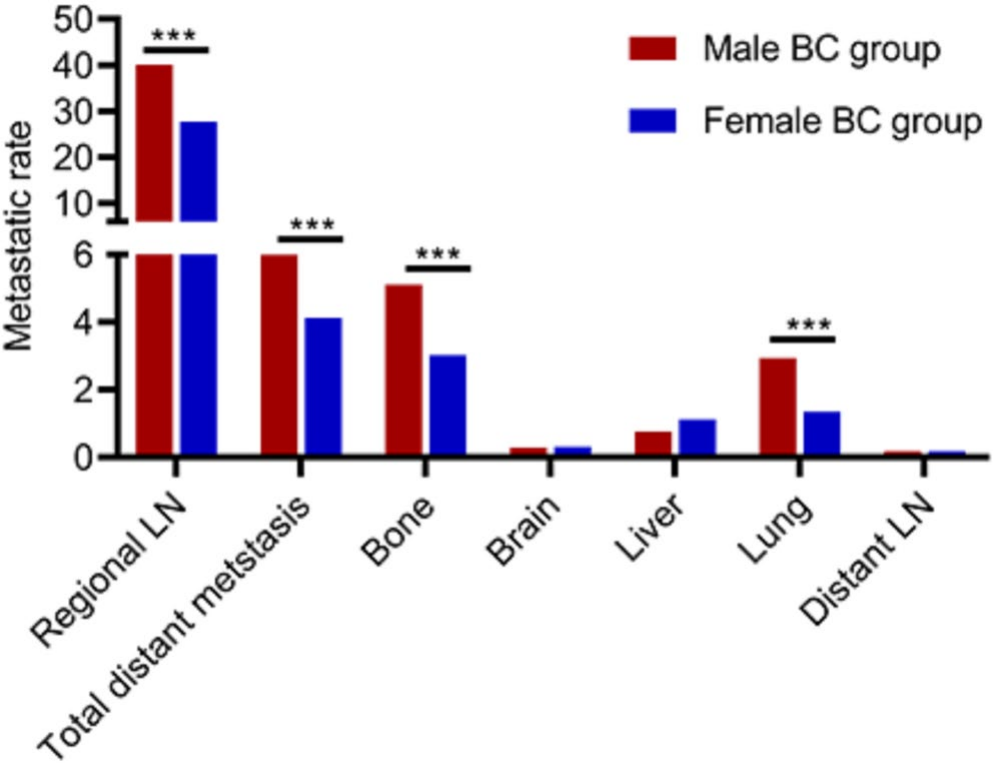
Comparison of the frequencies of different sites between male BC and female BC group

There were 683 patients (40.08%) in male group and 62,420 patients (27.69%) in female group who had positive regional LN when diagnosis. The regional LN metastatic rate of male BC was much higher than female BC (*p* < 0.05, Figure 1). When confounding variables such as age, race, marital status, histology, grade, laterality, and molecular subtype were adapted by multivariate analysis, the male BC group still had more regional LN metastasis [Hazards ratio (HR) 1.849, 95% confidence interval (CI) 1.674–2.043, *p* < 0.001, Table 2].

**TABLE 2:** Multivariate analyses of the impact of Mmale BC on different metastatic site

Variable	Metastatic site	OR	95%CI	*p* values
Male versus Female	Regional LN	1.849	(1.674–2.043)	*p *< 0.001
	Distant metastasis	1.421	(1.157–1.744)	0.001
	Bone	1.645	(1.320–2.051)	*p *< 0.001
	Lung	2.115	(1.587–2.819)	*p *< 0.001
	Liver	0.681	(0.393–1.179)	0.17
	Brain	1.011	(0.418–2.447)	0.981
	Distant LN	0.886	(0.284–2.768)	0.837

**FIGURE 2:**
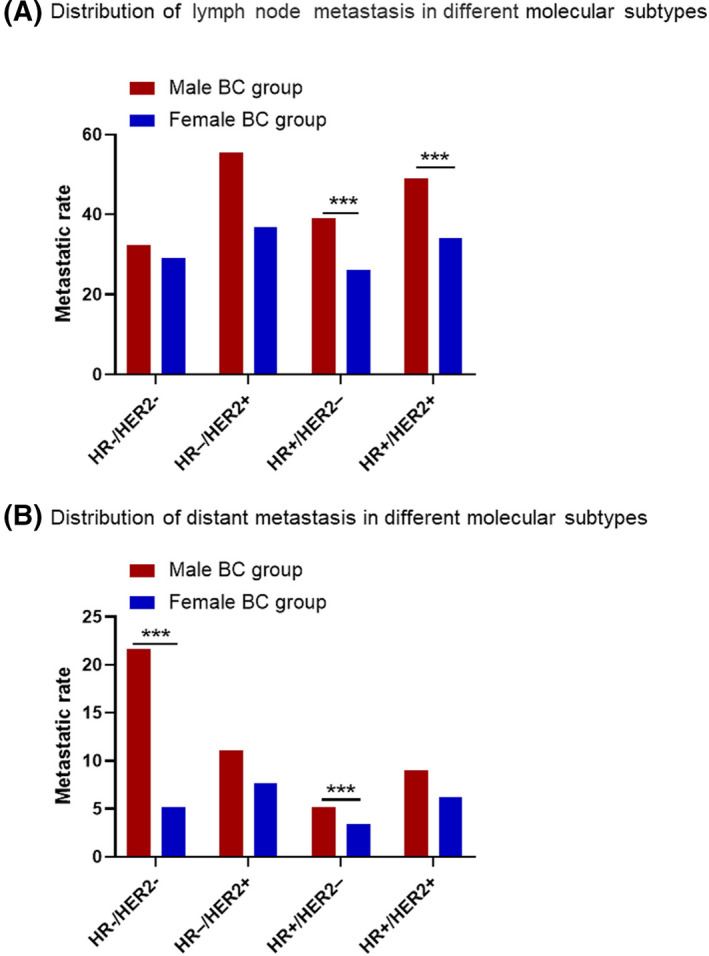
Distribution of lymph node (A) and distant (B) metastasis in different molecular subtypes

The affection of breast subtypes on regional LN metastasis was further explored in male BC and female BC group (Figure 2A). In either male BC group or female BC group, the regional LN metastatic rate was highest in HoR−/HER2+ subtype (male: 55.56%; female: 36.86%), followed by HoR+/HER2+ subtype. HoR−/HER− subtype had the lowest regional LN metastatic rate (32.43%) in male BC group, but it was HoR+/HER2‐ subtype (26.00%) in female BC group.

### 3.3 Distant metastasis

At the time of diagnosis, male BC cohort had a higher incidence of distant metastasis compared to the female group (5.93% vs. 4.12%, *p* < 0.001, Figure 1). When we adjusted confounding variables, the difference was still significant (HR 1.421, 95%CI: 1.157–1.744, *p* < 0.001, Table 2). The frequent metastatic lesions were bone, lung, liver, brain, and distant DL. Bone was the primary metastatic site, which accounted for 85.29% (87/102) of all distant metastasis in male BC group and 73.33% (6802/9276) in female BC group. Distant LN was the least frequent metastatic site, which accounted for 2.97% (3/101) in male BC group and 4.43% (411/9276) in female BC group. The incidence of bone metastasis and lung metastasis in male patients was significantly higher than female BC patients (*p* < 0.001, Figure 1). After adjusting confounding variables such as age, race, histology, and molecular subtype ect, it still showed that the male BC group had more bone metastasis (HR 1.645, 95%CI: 1.320–2.051, *p* < 0.001) and lung metastasis (HR 2.115, 95%CI: 1.587–2.819, *p* < 0.001) than female BC group (Table 2).

The affection of breast subtypes on distant metastasis was further explored in male BC and female BC group (Figure 2B). In the male BC group, HoR−/HER2− subtype had the highest distant metastatic rate (21.26%, Figure 2B). But in the female BC group, HoR−/HER2+ subtype had the highest distant metastatic rate (7.67%, Figure 2B). In either male BC group or female BC group, the distant metastatic rate was lowest in HoR+/HER2− subtype (Figure 2B).

### 3.4 Metastasis combinations

**FIGURE 3:**
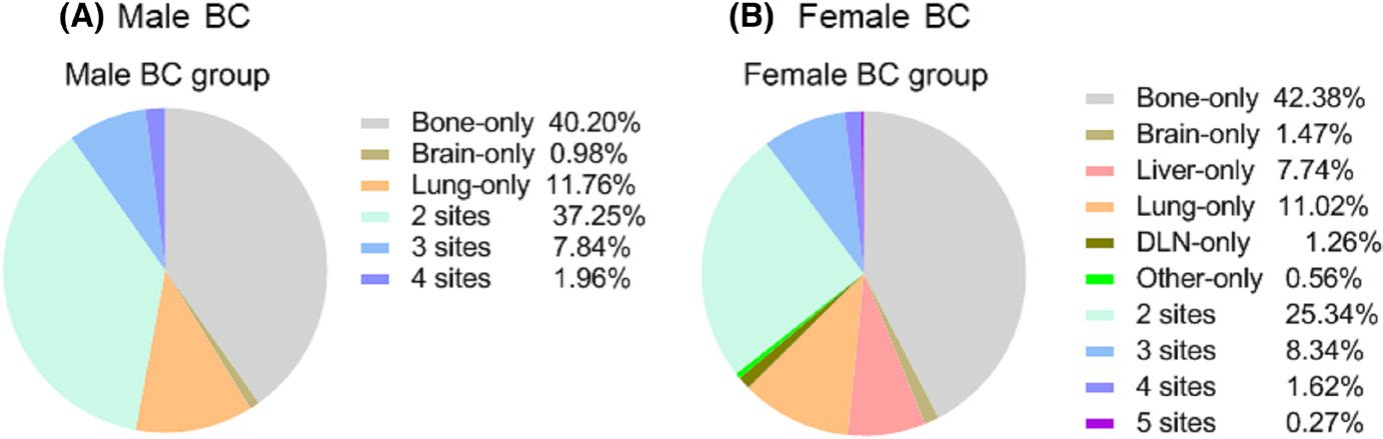
Relative rates of single‐organ and multiorgan metastatic sites in male BC (A) and female BC (B)

Massive patients show multiple organ metastasis when diagnosis. The relative rates of single‐organ and multi‐organ metastasis are shown in pie charts (Figure 3). For single‐site metastasis, bone (male: 40.20%, female: 42.38%) was the leading site and lung (male: 11.76%, female: 11.02%) was the second site in both male and female groups. For co‐metastasis, the bi‐gan pattern (male: 37.25%, female: 25.34%) revealed preponderance over the tri‐gan (male: 7.84%, female: 8.34%), tetra‐gan (male: 1.96%, female: 1.62%), and penta‐gan (male: 0.00%, female: 0.27%) patterns.

The frequencies of all possible combinations of the five metastatic lesions were compared between the two groups (Table 3). Bone was the most common single‐site metastasis (male: 2.41%, female: 1.74%, *p* = 0.038). The isolated liver metastasis between the two groups also showed great differences(male: 0.00%, female: 0.32%, *p* = 0.008). The most common bi‐site combination was the bone and lung (male: 1.58%, female: 0.40%, *p* = 0.000). The most common tri‐site metastasis was the bone, lung, and liver (male: 0.29%, female: 0.20%, *p* = 0.400). There were no distinct differences existed in all types of four sites and five sites metastatic combinations between male and female group.

**TABLE 3:** Frequencies of combined de novo metastases

Variable	Male BC (*n* = 1704)		Female BC (*n* = 225,417)		*p* values
	Number	(%)	Number	(%)	
One site					
Bone‐only	41	2.41	3931	1.74	0.038
Brain‐only	1	0.06	136	0.06	0.725
Liver‐only	0	0	718	0.32	0.008
Lung‐only	12	0.7	1022	0.45	0.125
DL‐only	0	0	117	0.05	1
Other‐only	0	0	52	0.02	1
Two sites					
Bone brain	2	0.12	174	0.08	0.381
Bone liver	6	0.35	794	0.35	1
Bone lung	27	1.58	907	0.4	0
Bone DL	1	0.06	62	0.03	0.374
Bone other	1	0.06	40	0.02	0.266
Brain liver	0	0	19	0.01	1
Brain lung	0	0	51	0.02	1
Brain DL	0	0	0	0	1
Brain other	0	0	1	0	1
Liver lung	0	0	238	0.11	0.432
Liver DL	0	0	13	0.01	1
Liver other	0	0	4	0	1
Lung DL	0	0	25	0.01	1
Lung other	1	0.06	7	0	0.058
DL other	0	0	16	0.01	1
Three sites					
Bone brain liver	0	0	66	0.03	1
Bone brain lung	1	0.06	99	0.04	0.529
Bone brain DL	0	0	3	0	1
Bone brain other	0	0	1	0	1
Bone liver lung	5	0.29	447	0.2	0.4
Bone liver DL	0	0	28	0.01	1
Bone liver other	0	0	16	0.01	1
Bone lung DL	0	0	38	0.02	1
Bone lung other	1	0.06	8	0	1
Bone DL other	0	0	20	0.01	1
Brain liver lung	0	0	21	0.01	1
Brain liver DL	0	0	2	0	1
Brain liver other	0	0	0	0	1
Brain lung DL	0	0	2	0	1
Brain lung other	0	0	1	0	1
Brain DL other	0	0	2	0	1
Liver lung DL	1	0.06	5	0	0.044
Liver lung other	0	0	2	0	1
Liver DL other	0	0	4	0	1
Lung DL other	0	0	9	0	1
Four sites					
Bone brain liver lung	1	0.06	99	0.04	0.529
Bone brain liver DL	0	0	1	0	1
Bone brain liver other	0	0	0	0	1
Bone brain lung DL	0	0	3	0	1
Bone brain lung other	0	0	1	0	1
Bone brain DL other	0	0	0	0	1
Bone liver lung DL	0	0	13	0.01	1
Bone liver lung other	0	0	4	0	1
Bone liver DL other	0	0	7	0	1
Bone lung DL other	1	0.06	16	0.01	0.12
Brain liver lung DL	0	0	0	0	1
Brain liver lung other	0	0	2	0	1
Brain liver DL other	0	0	1	0	1
Brain lung DL other	0	0	1	0	1
Liver lung DL other	0	0	2	0	1
Five sites					
Bone brain liver lung DL	0	0	7	0	1
Bone brain liver lung other	0	0	4	0	1
Bone brain liver DL other	0	0	2	0	1
Bone brain lung DL other	0	0	3	0	1
Bone liver lung DL other	0	0	8	0	1
Brain liver lung DL other	0	0	1	0	1

**FIGURE 4:**
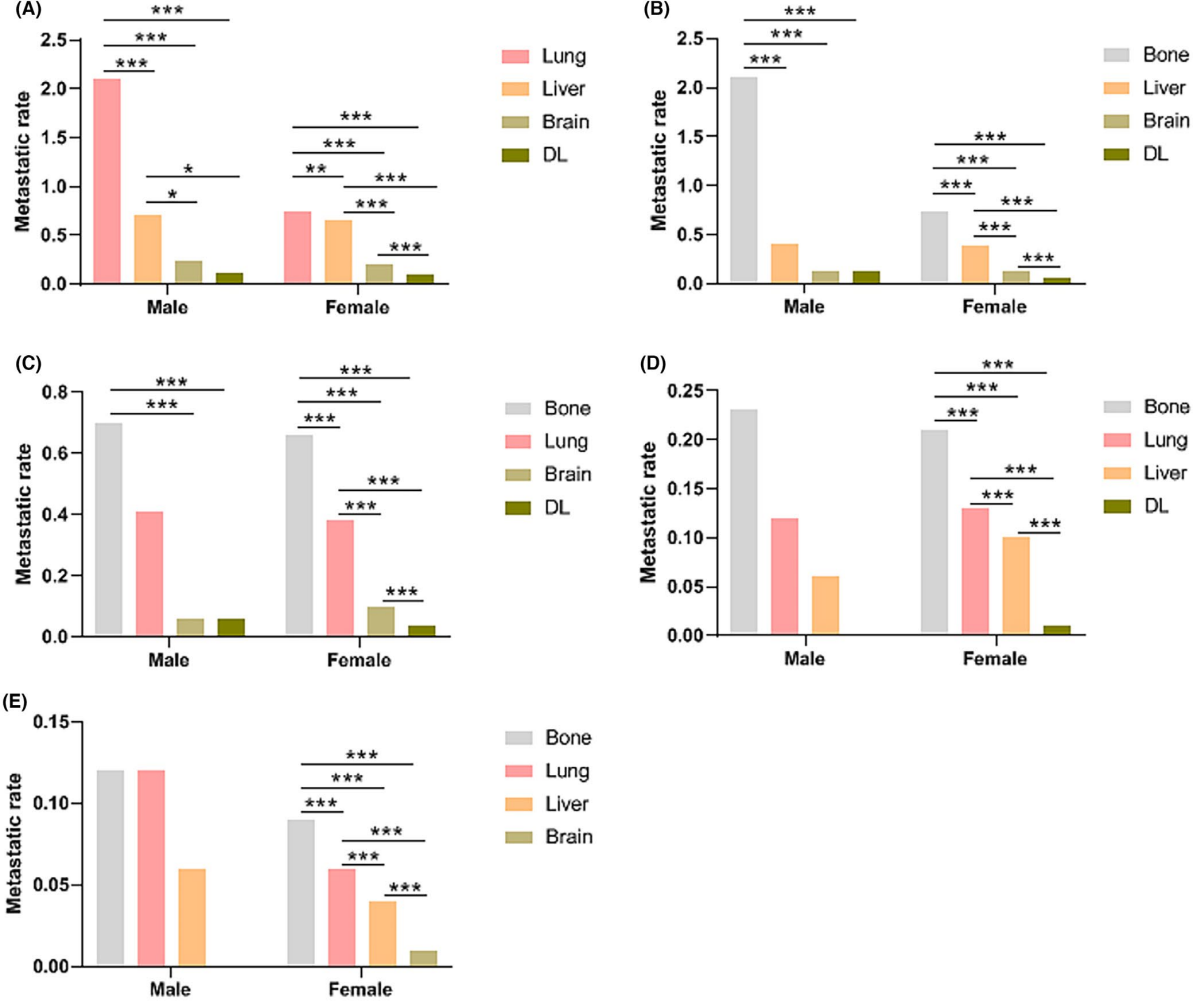
Comparisons of co‐metastatic rates in male BC and female BC. (A) Bone metastasis with other sites; (B) Lung metastasis with other sites; (C) Liver metastasis with other sites; (D) Brain metastasis with other sites; (E) DL metastasis with other sites

Moreover, we further analyzed the mutual effects among these metastasis (Figure 4A–E). Both male and female group had similar pattern of metastatic combinations. The incidence rate of bone metastasis was higher in patients with lung, liver, brain, or DL metastasis.

### 3.5 Survival

**FIGURE 5:**
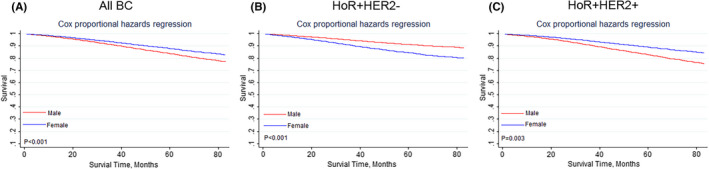
Kaplan–Meier curves of the impact of gender on overall survival in different molecular subtypes. (A)All breast cancer patients; (B) HoR+HER2− patient subtype; (C) HoR+HER2+ patient subtype

Our research observed 353 deaths in the male group (20.72%) and 26,839 deaths in the female group (11.82%). According to the Kaplan–Meier curves, male BC group had poorer OS than female BC cohort (Figure 5A, *p* < 0.001). Gender was as an independent prognostic factor for OS which was further suggested by the Cox regression analysis (HR 1.374, 95% CI 1.236–1.527, *p* < 0.001, Table 4). But considering the BCSD, male BC cohort had no significant difference from the female BC group (HR 1.049, 95% CI 0.869–1.266, *p* = 0.620, Table 5 and Figure S2A).

**TABLE 4:** Cox regression analyses for OS

Clinicopathological characteristics	Hazard ratio (95%CI)	*p* values
Gender		
Female	Reference	
Male	1.374(1.236–1.527)	<0.001
Age		
<=60	Reference	
60–70	1.384(1.337–1.433)	<0.001
>70	3.001(2.910–3.094)	<0.001
Race		
White	Reference	
Black	1.195(1.153–1.239)	<0.001
Others	0.766(0.734–0.800)	<0.001
Laterality		
Right	Reference	
Left	0.992(0.969–1.016)	0.513
Other	0.881(0.766–1.014)	0.078
Marital status		
Married	Reference	
Unmarried	1.401(1.365–1.438)	<0.001
Unknown	1.187(1.126–1.251)	<0.001
Histology		
Ductal	Reference	
Lobular	1.027(0.986–1.070)	0.2
Other	1.045(1.008–1.083)	0.016
Grade		
Ⅰ	Reference	
Ⅱ	1.169(1.125–1.215)	<0.001
Ⅲ/Ⅳ	1.714(1.644–1.788)	<0.001
Unknown	1.293(1.221–1.369)	<0.001
AJCC T classification		
T1	Reference	
T2	1.607(1.557–1.658)	<0.001
T3	2.189(2.091–2.293)	<0.001
T4	2.598(2.476–2.725)	<0.001
Other	1.643(1.547–1.744)	<0.001
AJCC N classification		
N0	Reference	
N1	1.346(1.304–1.389)	<0.001
N2	1.955(1.865–2.049)	<0.001
N3	2.345(2.233–2.463)	<0.001
Unknown	1.555(1.459–1.657)	<0.001
AJCC M classification		
M0	Reference	
M1	2.489(2.395–2.588)	<0.001
Unknown	0.694(0.616–0.782)	<0.001
Molecular subtype		
HoR−/HER2−	Reference	
HoR−/HER2+	0.565(0.0.533–0.598)	<0.001
HoR+/HER2−	0.486(0.469–0.504)	<0.001
HoR+/HER2+	0.454(0.433–0.476)	<0.001
Surgery		
Yes	Reference	
No/unknown	0.337(0.325–0.349)	<0.001
Chemotherapy		
Yes	Reference	
No/unknown	0.667(0.649–0.685)	<0.001
Radiation therapy		
Yes	Reference	
No/unknown	0.725(0.703–0.747)	<0.001

**TABLE 5:** Competing risk analyses for BCSD

Clinicopathological characteristics	Hazard ratio (95%CI)	*p* values
Gender		
Female	Reference	
Male	1.049(0.869–1.266)	0.62
Age		
<=60	Reference	
60–70	1.009(0.960–1.060)	0.733
>70	1.327(1.262–1.396)	<0.001
Race		
White	Reference	
Black	1.235(1.167–1.306)	<0.001
Others	0.843(0.791–0.899)	<0.001
Laterality		
Right	Reference	
Left	0.999(0.961–1.038)	0.945
Other	0.873(0.703–1.085)	0.221
Marital status		
Married	Reference	
Unmarried	1.231(1.181–1.283)	<0.001
Unknown	1.209(1.110–1.316)	0.205
Histology		
Ductal	Reference	
Lobular	1.126(1.052–1.205)	0.001
Other	1.009(0.949–1.073)	0.776
Grade		<0.001
Ⅰ	Reference	
Ⅱ	1.712(1.578–1.857)	<0.001
Ⅲ/Ⅳ	2.985(2.740–3.251)	<0.001
Unknown	2.162(1.946–2.401)	<0.001
AJCC T classification		
T1	Reference	
T2	2.330(2.197–2.471)	<0.001
T3	3.381(3.137–3.644)	<0.001
T4	3.743(3.449–4.062)	<0.001
Other	2.460(2.227–2.718)	<0.001
AJCC N classification		
N0	Reference	
N1	1.968(1.868–2.073)	<0.001
N2	2.832(2.637–3.041)	<0.001
N3	3.224(2.985–3.481)	<0.001
Unknown	1.940(1.742–2.161)	<0.001
AJCC M classification		
M0	Reference	
M1	3.238(3.054–3.434)	<0.001
Unknown	0.740(0.619–0.885)	0.001
Molecular subtype		
HoR−/HER2−	Reference	
HoR−/HER2+	0.511(0.469–0.557)	<0.001
HoR+/HER2−	0.465(0.440–0.492)	<0.001
HoR+/HER2+	0.407(0.379–0.437)	<0.001
Surgery		
Yes	Reference	
No/unknown	0.364(0.344–0.386)	<0.001
Chemotherapy		
Yes	Reference	
No/unknown	0.906(0.863–0.953)	<0.001
Radiation therapy		
Yes	Reference	
No/unknown	0.914(0.876–0.952)	<0.001

Since most male BC patients had positive HoR expression, we further analyzed the impact of gender on OS and BCSD in HoR positive patients. Cox regression analysis revealed that male BC cohort had poorer OS than the female BC group in both HoR+/HER2− (Figure 5B, *p* < 0.001) and HoR+/HER2+ group (Figure 5C, *p* = 0.003). But competing risk model showed male BC cohort had similar cumulative incidence of BCSD as the female BC group in both HoR+/HER2− (Figure S2B, *p* = 0.606) and HoR+/HER2+ group (Figure S2C, *p* = 0.277).

**FIGURE 6:**
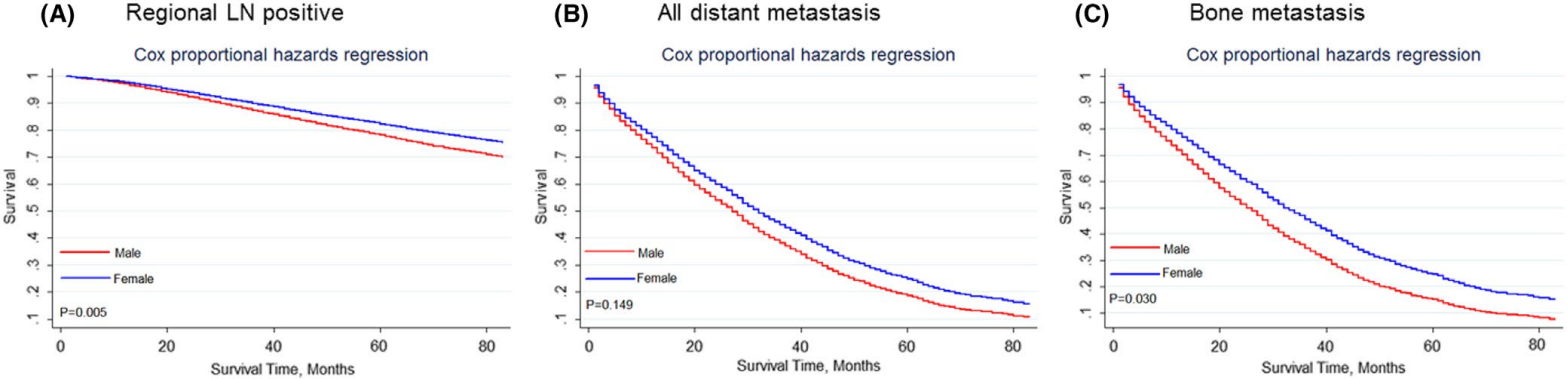
Kaplan–Meier curves of the impact of gender on overall survival in different metastatic subgroups. (A) Regional LN positive subgroup; (B) Distant metastasis positive subgroup; (C) Bone metastasis positive subgroup

We further investigated the impact of gender on the survivorship of patients with metastasis. In the subgroup of patients with positive regional LN, the male BC group had worse OS than the female BC group (Figure 6A, *p* = 0.005). But competing risk analysis showed that gender had no significant effect on the cumulative incidence of BCSD in this subgroup (Figure S3A, *p* = 0.379). Subgroup analysis also revealed that the male BC group had similar OS (Figure 6B, *p* = 0.149) and cumulative incidence of BCSD (Figure S3B, *p* = 0.862) as the female BC group in patients with all distant metastasis. Bone was the most frequent distant metastatic site of BC,^24^ hence we further did survival analysis in this subgroup. The male BC group with bone metastasis had worse OS (Figure 6C, *p* = 0.030) than female BC group with bone metastasis, but BCSD was not significantly higher (Figure S3C, *p* = 0.678).

To investigate further, PSM analysis was performed to adjust for the unmatching cohort, and a total of 1666 female BC patients were matched with 1666 male BC patients (1:1). After PSM, the clinicopathological characteristics between male BC group and female BC group showed no significantly difference (Table S1). On this basis, we performed the Kaplan–Meier analysis. Likewise, the result showed that male BC group had poorer OS (Figure S4A, *p* < 0.001) but similar BCSD (HR 1.169, 95% CI 0.892–1.533, *p* = 0.256, Figure S4B) with female BC cohort.

## 4 DISCUSSION

In our research, we comprehensively analyzed and compared the metastatic patterns and prognosis between male and female BC through the SEER database. The results indicated that male BC had a higher rate in regional LN metastasis and distant metastasis, poorer OS, but similar BCSD. Besides, the affection of breast molecular subtypes on regional LN metastasis and distant metastasis in male and female cohorts was different. Gender was an independent prognostic factor for OS but not for BCSD in the general population. In the subgroup of patients with HoR+/HER2−, HoR+/HER2+, regional LN positive, or bone metastasis, the male cohort had poorer OS but similar BCSD with female cohort.

In our study, male patients presented with malignancy that were larger than female, and were prone to have LN and distant metastasis. Bone was the leading site of metastasis and lung was the second frequent site of metastasis in both male and female groups. The incidence of bone metastasis and lung metastasis in male patients was much higher than those in female BC patients. According to existing research results, men presenting with more advanced disease mainly because low public consciousness and the lack of screening procedures delay the best diagnosis time.^1^ Within 3 months of symptom onset, only less than half of male BC patients would be confirmed the disease compared with female BC patients, reported by *Rudan* et al.^25^ Without separating out the patients whose disease were diagnosed by breast screening and whose disease were diagnosed by active consultation, it is probably fair to say that comparing neoplasm inherent characteristics between male and female is not completely scientific. Unfortunately, cancer registry data in SEER database cannot allow us to make that distinction.^3^ In addition, underlying genetic and epigenetic differences also lead to the tumor stage discrepancy. For example, DNA‐repaired genes CHEK2^26^, ^27^ or PALB2^28^ are associated with metastatic male BC. Another study found that the T to C substitution^29^ in the promoter region related to increased risk of BC in men but not in women. As far as we are concerned, except the above reasons, physiology differences especially endocrine system differences may also cause this phenomenon. Cancer is ultimately triggered by the failure of immune surveillance. The divergent effects of estrogen and androgens on antitumor immunity could result in the poorer outcome in men,^30^, ^31^ BC is no exception. Despite great progress has been made in tumor immunology recently, currently our comprehension of the crosstalk between sex hormones and antitumor immunological effects is still in its infancy. Thus, the effect sex hormones have on differences in male BC and female BC need to be further explored.

The molecular subtype classification system, which was established by gene expression study, has demonstrated prognostic significance,^11^, ^32^ therefore it has been influencing BC management over the past decades. In our study, the affection of breast molecular subtypes on metastasis was further explored in male BC group and female BC group. It is worth mentioning that the time node of metastasis listed in our study was before clinical interventions. In either male BC group or female BC group, HoR−/HER2+ subtype had the highest regional LN metastatic rate, HoR+/HER2+ subtype had the second highest regional LN metastatic rate. HoR−/HER− subtype had the lowest regional LN metastatic rate in male BC group, but in female BC group, HoR+/HER2− subtype had the lowest regional LN metastatic rate. To sum up, HER+ subtype have a higher regional LN metastatic rate than HER‐ subtype. The increasing interest in minimizing axillary surgery^33^ is clearly evident with the advancement of adjuvant therapy. Still, surgical staging is routinely performed in all patients with negative lymph node clinically, despite the truth that majority of them have tumor‐free axilla.^34^, ^35^ For these patients, invasive surgery brings no treatment benefit but possible clinical complications. Our study might give the hint that for those clinically node‐negative patients whose biopsy pathology confirmed as HER2+ subtype, surgical axillary staging still cannot be undervalued, but HER2− subtype especially HoR−/HER− subtype in male patients and HoR+/HER2− subtype in female patients may obtain an axillary surgery exemption in the future. Certainly, speculation mentioned above still need more clinical randomized controlled trials to be further confirmed. Our study further proved that, in the male BC group, HoR−/HER2− subtype had the highest distant metastatic rate, but in the female BC group, HoR−/HER2+ subtype had the highest metastatic rate. It is generally known that BC molecular subtype was independent factors affecting the emergence of metastasis.^8^, ^36^ And majority of the BC show hormone receptor expression^37^ in both male and female patients. HoR−/HER− male BC is not only rare, but also reported to have a notably stronger invasiveness than female HoR−/HER2− or other BC subtypes,^38^ which may explain its high rate in distant metastasis. Compared with HoR−/HER2− subtype and HER2+ subtype, luminal subtype showed innate inertness in either male or female BC groups,^37^ thus made up the minority of the distant metastasis. This is consistent with our research result that in either male BC group or female BC group, HoR+/HER2− subtype had the lowest distant metastatic rate. Admittedly, underlying genetic differences in tumor biology is the root drive factor that causes metastasis differences^39^ between male and female groups. Johansson and his colleagues found that only two driver genes were common between male and female patients while analyzed more than 100 comparative genome hybridization data.^40^ More fundamental researches are needed to dig more tumor cell intrinsic molecular mechanisms to guide the recognition of clinical male BC.

Many patients showed multi‐organ metastasis at initial diagnosis. Next, the frequencies of all possible combinations of the five metastatic lesions were compared between the two groups. Bone was the most common single‐site metastasis. The most common bi‐site combination was the bone and lung. The most common tri‐site metastasis was the bone, lung, and liver. By contrast, except the single liver metastasis was lower in male BC patients, single‐site metastasis rate and bi‐site metastasis rate were both higher in male patients than in female patients. There were no distinct differences existed in all types of four sites and five sites metastatic combinations between male and female group. According to research reported by Li et al, 90% of male BC are hormone positive, and usually luminal subtype tumors have a predilection for skeleton metastases.^41^ Thus, the highest incidence of bone metastasis rates was consistent with previous study. Likewise, HER2 overexpression may be closely involved in the seeding of the liver parenchyma,^8^ yet HER2+ subtype is more likely to be negative in male BC. This may explain the lower occurrence rate of liver metastasis in male BC than in female BC. After analyzed the interactions among these metastatic lesions, we also found that the incidence rate of bone metastasis was higher in patients with lung, liver, brain, or DL metastasis. This requires clinicians to pay attention to the possibility of combined metastasis in different sites and especially be more aware of bone metastasis after one single metastasis site was diagnosed.

Several studies have evaluated survivorship in male BC before,^2^, ^42^ which were consistent with our research results. The male BC group had poorer OS than female BC cohort, and the gender was an independent prognostic factor for OS. But considering the BCSD, male BC cohort had no significant difference from the female BC group, the gender failed to be an independent prognostic factor for BCSD. Since most male BC patients had positive HoR expression, we further confirmed the conclusion above in both HoR+/HER2− and HoR+/HER2+ group. Because the small sample size (only 18 male patients were HoR−/HER2− subtype and 37 male patients were HoR−/HER2+) would lead to the statistical limitations of the study, we failed to conduct the Cox regression analyses and competing risk model in HoR−/HER2− and HoR−/HER2+ subtypes. The results above demonstrate that despite the male group tend to have a more advanced stage of tumor and an older age at diagnosis, the BCSD had not increased. This mainly can be explained by that 95% of male BC were hormone positive which is more curable than HER2+ or HoR−/HER2− subtype. The advancement of adjuvant therapy further enabled to prolong the life expectancy in male patients. Meanwhile, the poorer OS corroborated the shorter life expectancy in men than in women.^43^

Metastastic lesions often contributes to the bad outcome of patients. We further investigated the influence of gender on the survivorship of patients with metastasis. In the subgroup of patients with positive regional LN, male BC had worse OS than female BC, but the cumulative incidence of BCSD was not increased. Nowadays, regional LN metastasis especially 1–2 positive axillary nodes was not enough to pose a threat to long‐term survivorship. Therefore, the clinical trial ACOSOG Z0011 recommend those patients with T1‐2 primary tumors, with clinically negative axilla, with 1–2 positive sentinel lymph nodes, with undergoing breast‐conserving surgery and adjuvant whole‐breast irradiation are able to abandon the axillary lymph node dissection.^44^ Similar cumulative incidence of BCSD between male and female corhots may imply that the result of ACOSOG Z0011 may also be applied to male patients. Subgroup analysis also revealed that the male BC group had similar OS and cumulative incidence of BCSD as the female BC group in patients with all distant metastasis. Bone was the most frequent distant metastatic site of BC. The male BC group with bone metastasis had worse OS but similar BCSD with female BC group with bone metastasis. In general population, we found that male BC patients had poorer OS as mentioned above, but focused on metastatic patients (except patients with bone metastasis), there were no differences in survival between male and female patients. Also, BCSD was not affected by gender. These results might indicated that more nonmetastatic early male BC patients or single‐bone metastatic male patients passed due to other comorbidities compared with corresponding type of female patients. It is generally known that there exists a sexual dimorphism in human life expectancy, and men exhibit shorter life expectancy than women.^43^ When early‐staged or single‐bone metastastic BC is not sufficient to kill patients quickly, comorbidities shortening the human life span then play a leading role in affecting OS in patients. Multiple prospective and retrospective study have showed that male patients present with greater Charlson‐Deyo comorbidity score than females, specifically include coronary artery disease, chronic lung disease, cerebrovascular disease, peripheral vascular disease etc.^45^, ^46^, ^47^, ^48^ Therefore, comorbidity assessment and management should be more integrated into treatment decisions especially in nonmetastatic or single‐bone metastastic male BC patients.

We recognize that our research has some inadequacies. First, the information of other distant metastatic lesion such as pleura, peritoneum has not been collected by SEER database currently, which limit the overall prognostic assessment. Furthermore, the metastasis information in SEER database is only collected at initial diagnosis, future analysis needs to cover longer follow‐up details. Last but not least, despite we had performed the multifactorial regression analysis, there still existed a selection bias as this analysis was a retrospective research.

## 5 CONCLUSION

Conclusionly, male BC had different metastasis patterns and prognostic outcomes compared with female patients. Besides, the affection of breast subtypes on metastasis and survivorship in male and female cohorts was different. Thus, in clinical work, we should deal with different molecular subtypes differently and customized more personalized therapeutic strategies for male patients rather than simply copy the clinical experience in women. Furthermore, gender was an independent prognostic factor for OS, but not for BCSD. However, in the subgroup of distant metastasis, the gender no longer had any impact on survivorship. Therefore, metastatic disease must be given sufficient attention and more effective management should be explored for metastatic BC.

## ETHICS STATEMENT

This study complied with the Declaration of Helsinki. The ethics committee of The First Affiliated Hospital of Nanjing Medical University exempted the patient informed consent, because there was no personal identification applied and no direct interaction with patients in this study.^13^ Data use agreement of SEER database was assigned.

## CONFLICT OF INTEREST

The authors declare that there is no conflict of interest.

## Supporting information

Figure S1

Figure S2

Figure S3

Figure S4

Table S1

## Data Availability

The data that support the findings of this study are publicly available in National Cancer Institute's Surveillance, Epidemiology, and End Results (SEER) program 1975 to 2016 Research Plus Additional Custom Treatment Data (www.seer.cancer.gov).
