# Supplementary figures and images for: Characteristics of metastasis and survival between male and female breast cancer with different molecular subtypes: A population‐based observational study
Source: Cancer Med. 2021 Dec 12;11(3):764–77. doi: 10.1002/cam4.4469 (PMC8817100; doi:10.1002/cam4.4469)

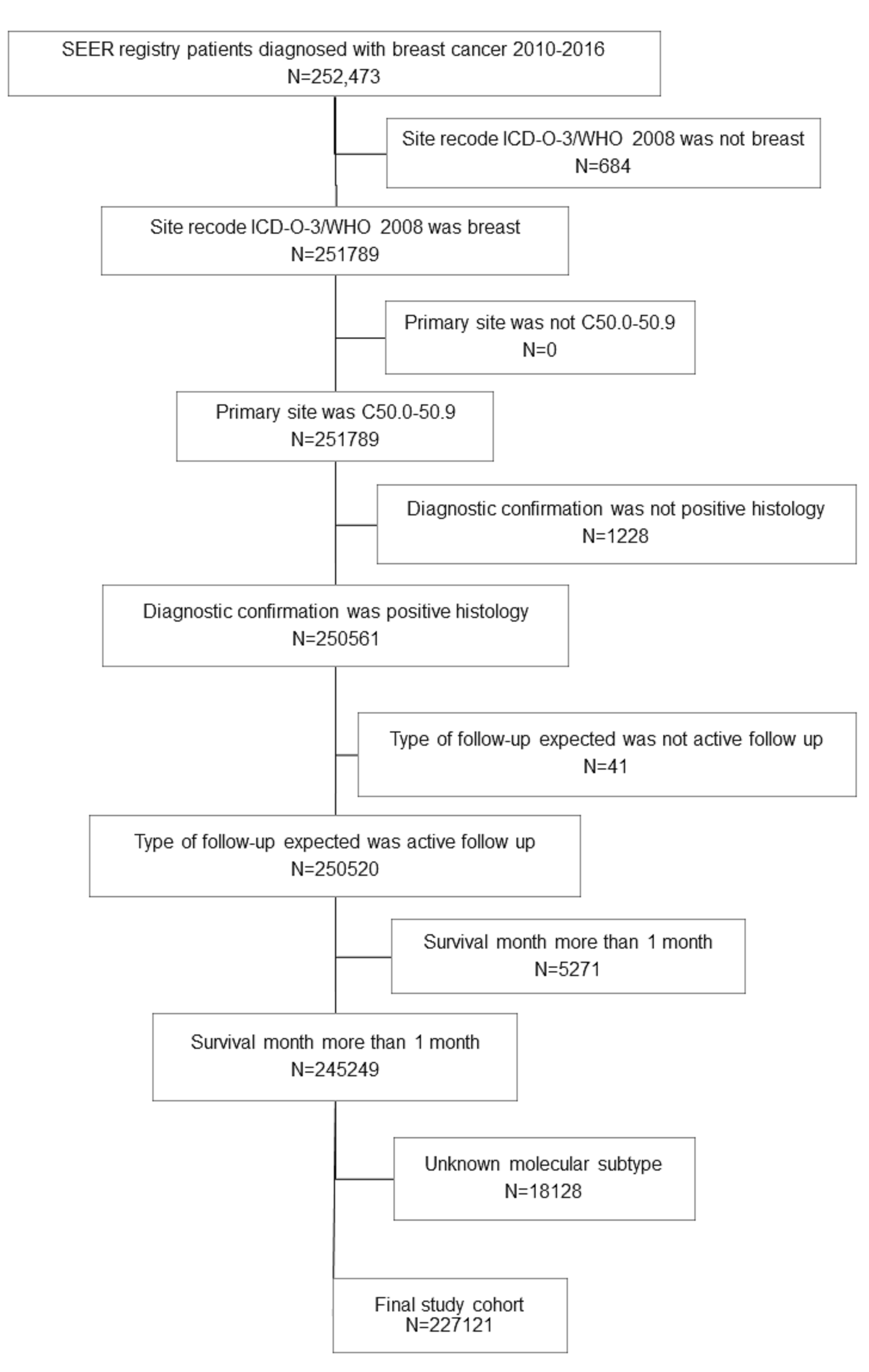

Supplement: Supplementary file 1 — Figure S1 [file CAM4-11-764-s003.jpeg]

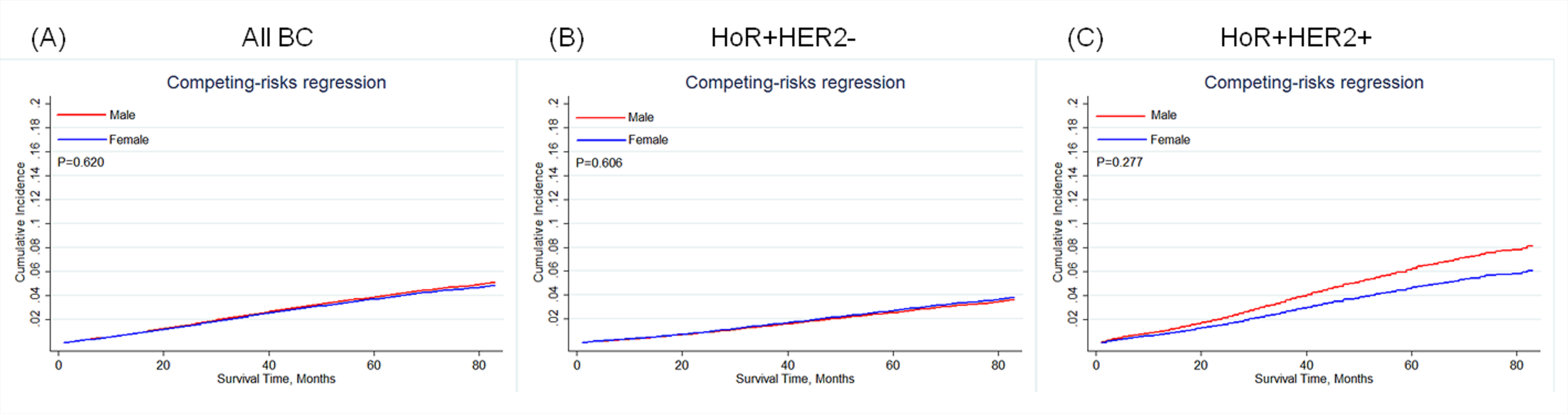

Supplement: Supplementary file 2 — Figure S2 [file CAM4-11-764-s002.tif]

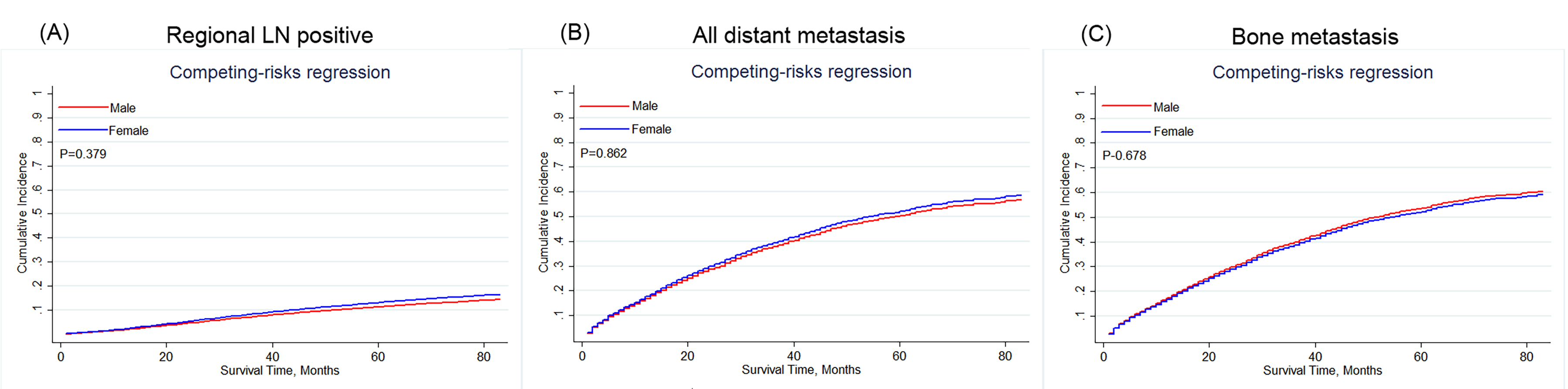

Supplement: Supplementary file 3 — Figure S3 [file CAM4-11-764-s001.tif]

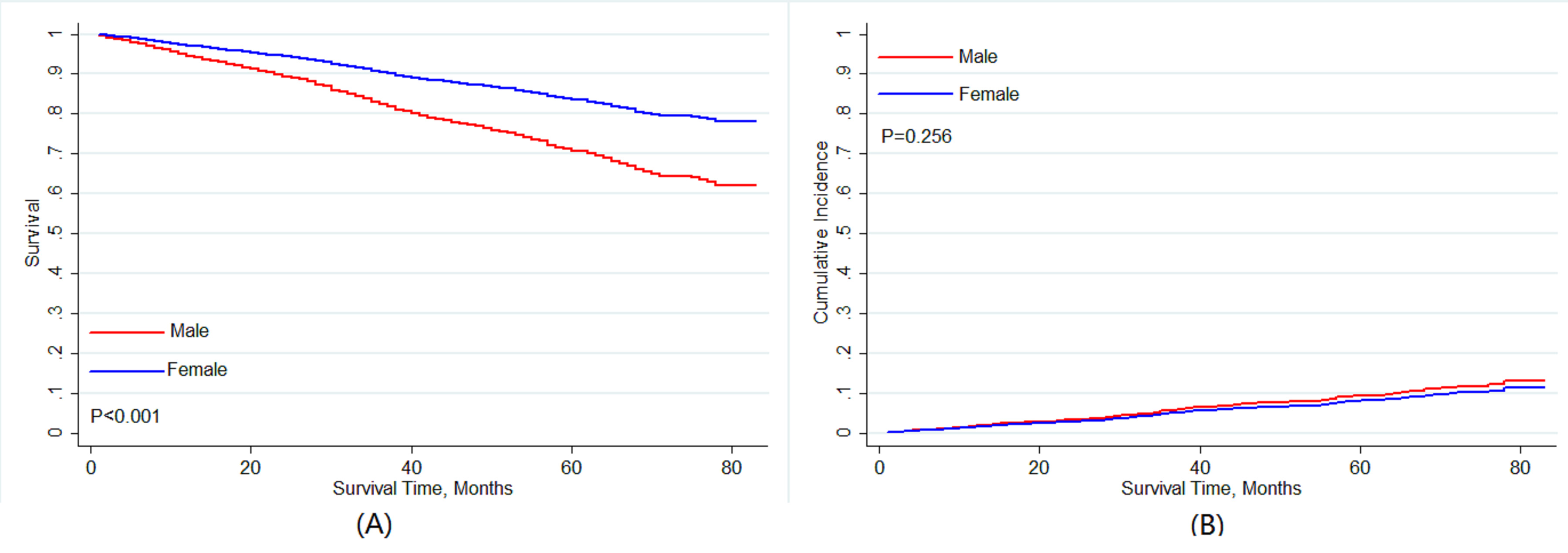

Supplement: Supplementary file 4 — Figure S4 [file CAM4-11-764-s004.jpeg]
