# Supplementary material for: Characteristics of metastasis and survival between male and female breast cancer with different molecular subtypes: A population‐based observational study
Source: Cancer Med. 2021 Dec 12;11(3):764–77. doi: 10.1002/cam4.4469 (PMC8817100; doi:10.1002/cam4.4469)
Supplement: Supplementary file 5 — Table S1 [file CAM4-11-764-s005.docx]

| **Table S1 Baseline clinical characteristics of male BC and female BC after propensity score matching in the SEER database** | | | | |  |
| --- | --- | --- | --- | --- | --- |
| **Characteristics** | | **Male BC(n=1666)** | **Female BC(n=1666)** | **P values** |  |
|  |  |  |  |  |  |
| **Age** |  |  |  |  |  |
|  | ≤60 | 450 | 455 | 0.846 |  |
|  | >60 | 1216 | 1211 |  |  |
| **Race** |  |  |  |  |  |
|  | White | 1311 | 1310 | 0.966 |  |
|  | Other | 355 | 356 |  |  |
| **Laterality** |  |  |  |  |  |
|  | Right | 787 | 788 | 0.972 |  |
|  | Other | 879 | 878 |  |  |
| **Marital status** |  |  |  |  |  |
|  | Married | 1080 | 1086 | 0.827 |  |
|  | Unmarried /Unknown | 586 | 580 |  |  |
| **Grade** |  |  |  |  |  |
|  | Ⅰ/Ⅱ | 1059 | 1064 | 0.857 |  |
|  | Ⅲ/Ⅳ /Unknown | 607 | 602 |  |  |
| **AJCC stage** |  |  |  |  |  |
|  | Ⅰ | 499 | 499 | 1 |  |
|  | Ⅱ | 597 | 594 |  |  |
|  | Ⅲ | 227 | 227 |  |  |
|  | Ⅳ | 84 | 83 |  |  |
|  | Unknown | 259 | 263 |  |  |
| **Molecular subtype** |  |  |  |  |  |
|  | HoR+/HER2- | 177 | 182 | 0.988 |  |
|  | HoR+/HER2+ | 1443 | 1438 |  |  |
|  | HoR-/HER2+ | 12 | 13 |  |  |
|  | HoR-/HER2- | 34 | 33 |  |  |
| **Surgery** |  |  |  |  |  |
|  | Yes | 1526 | 1527 | 0.95 |  |
|  | No/unknown | 140 | 139 |  |  |
| **Chemotherapy** |  |  |  |  |  |
|  | Yes | 604 | 610 | 0.829 |  |
|  | No/unknown | 1062 | 1056 |  |  |
| **Radiation therapy** |  |  |  |  |  |
|  | Yes | 470 | 466 | 0.877 |  |
|  | No/unknown | 1196 | 1200 |  |  |
